# The accumulation of microplastic pollution in a commercially important fishing ground

**DOI:** 10.1038/s41598-022-08203-2

**Published:** 2022-03-10

**Authors:** Eoghan M. Cunningham, Sonja M. Ehlers, Konstadinos Kiriakoulakis, Pia Schuchert, Nia H. Jones, Louise Kregting, Lucy C. Woodall, Jaimie T. A. Dick

**Affiliations:** 1 Queen’s University Marine Laboratory, Queen’s University Belfast, 12-13 The Strand, Portaferry, BT22 1PF Northern Ireland UK; 2 School of Biological and Environmental Sciences, Liverpool John Moores University, 3 Byrom St, Liverpool, L3 3AF UK; 3 Department of Zoology, University of Oxford, 11a Mansfield Road, Oxford, OX1 3SZ UK; 4 Department of Animal Ecology, Federal Institute of Hydrology, Am Mainzer Tor 1, 56068 Koblenz, Germany; 5 Institute for Integrated Natural Sciences, University of Koblenz-Landau, 56070 Koblenz, Germany; 6 Agri-Food and Biosciences Institute, 18a Newforge Lane, Belfast, BT9 5PX Northern Ireland UK; 7 School of Ocean Sciences, Bangor University, Anglesey, LL59 5AB UK; 8 School of Natural and Built Environment, Queen’s University Belfast, Belfast, BT9 5BN UK; 9 Nekton Foundation, Oxford, OX5 1PF UK

**Keywords:** Conservation biology, Marine biology, Physical oceanography, Environmental impact

## Abstract

The Irish Sea is an important area for Norway Lobster *Nephrops norvegicus* fisheries, which are the most valuable fishing resource in the UK. Norway lobster are known to ingest microplastic pollution present in the sediment and have displayed reduced body mass when exposed to microplastic pollution. Here, we identified microplastic pollution in the Irish Sea fishing grounds through analysis of 24 sediment samples from four sites of differing proximity to the Western Irish Sea Gyre in both 2016 and 2019. We used µFTIR spectroscopy to identify seven polymer types, and a total of 77 microplastics consisting of fibres and fragments. The mean microplastics per gram of sediment ranged from 0.13 to 0.49 and 0 to 1.17 MP/g in 2016 and 2019, respectively. There were no differences in the microplastic counts across years, and there was no correlation of microplastic counts with proximity to the Western Irish Sea Gyre. Considering the consistently high microplastic abundance found in the Irish Sea, and the propensity of *N. norvegicus* to ingest and be negatively impacted by them, we suggest microplastic pollution levels in the Irish Sea may have adverse impacts on *N. norvegicus* and negative implications for fishery sustainability in the future.

## Introduction

The production of plastic has now reached record levels, with > 350 million tons produced in 2019^1^. This growth coincides with consumer demand in an ever-growing world population, where plastic products are utilised daily. A lack of suitable waste management has led to an estimated 10% of total plastic production entering marine environments every year^2^. As a result, plastic pollution is now a common feature of marine systems and has been found in remote areas such as the Polar Regions^3^ and the deep-sea^4^. To date, few studies have investigated the presence of plastic pollution in sediment from commercial fishing grounds, which is critical to understand the relative importance of microplastics as a threat for sustainable fisheries.

The formation of secondary microplastic particles (< 5 mm) through the fragmentation of larger sized plastics in marine systems, and the input of primary (i.e. manufactured) microplastics from terrestrial and aquatic systems is now a major environmental concern^5,6^. Owing to microplastic ubiquity in marine systems and the wide variety of feeding mechanisms in marine organisms, the ingestion of microplastics has been documented across many taxa^7^, with associated adverse impacts ranging from organ damage to reduced energy levels^8^. Negative impacts have also been found in commercially important fishery species bound for human consumption; including reduced body mass in crustaceans^9^, and reduced byssus thread production in bivalves^10^.

The Irish Sea, located between the islands of Ireland and Great Britain, is an important body of water for regional trade, shipping, and commercial fishing^11^. With its location between the UK and Ireland, the Irish Sea is also a valuable asset for mainland European economies due to the export of fishery species^12^. A number of commercially important species reside on the benthic sediment of the Irish Sea, including the Norway lobster (*Nephrops norvegicus*) and haddock (*Melanogrammus aeglefinus*), however, *N. norvegicus* is the primary target and accounts for 90% of the total Irish Sea landings^13^. It is also a critically important species for European fisheries, contributing to ~ 11% of the total European landings^12^.

As an endobenthic species, *N. norvegicus* burrows into the bottom sediment, with females remaining in their burrows for long durations (9–13 months) to incubate their eggs^14^. Marine sediment is a known sink for microplastic pollution^15^, and as a result, a range of decapod species are exposed to microplastics in the wild^16,17^, including Irish Sea species such as *N. norvegicus*^9^. A recent study documented that an average of 1.75 ± 2.01 microplastic particles were found in the gut of *N. norvegicus*^18^, most likely stemming from microplastics in the surrounding sediment or via trophic transfer^9^. A previous study also showed that the ingestion of low levels of microplastics reduced the body mass of *N. norvegicus*^9^. A reduced body mass, as a result of microplastic ingestion, has the potential to interfere with minimum landing sizes (MLS) and therefore reduce the sustainability of the fishery^14^.

**Figure 1:**
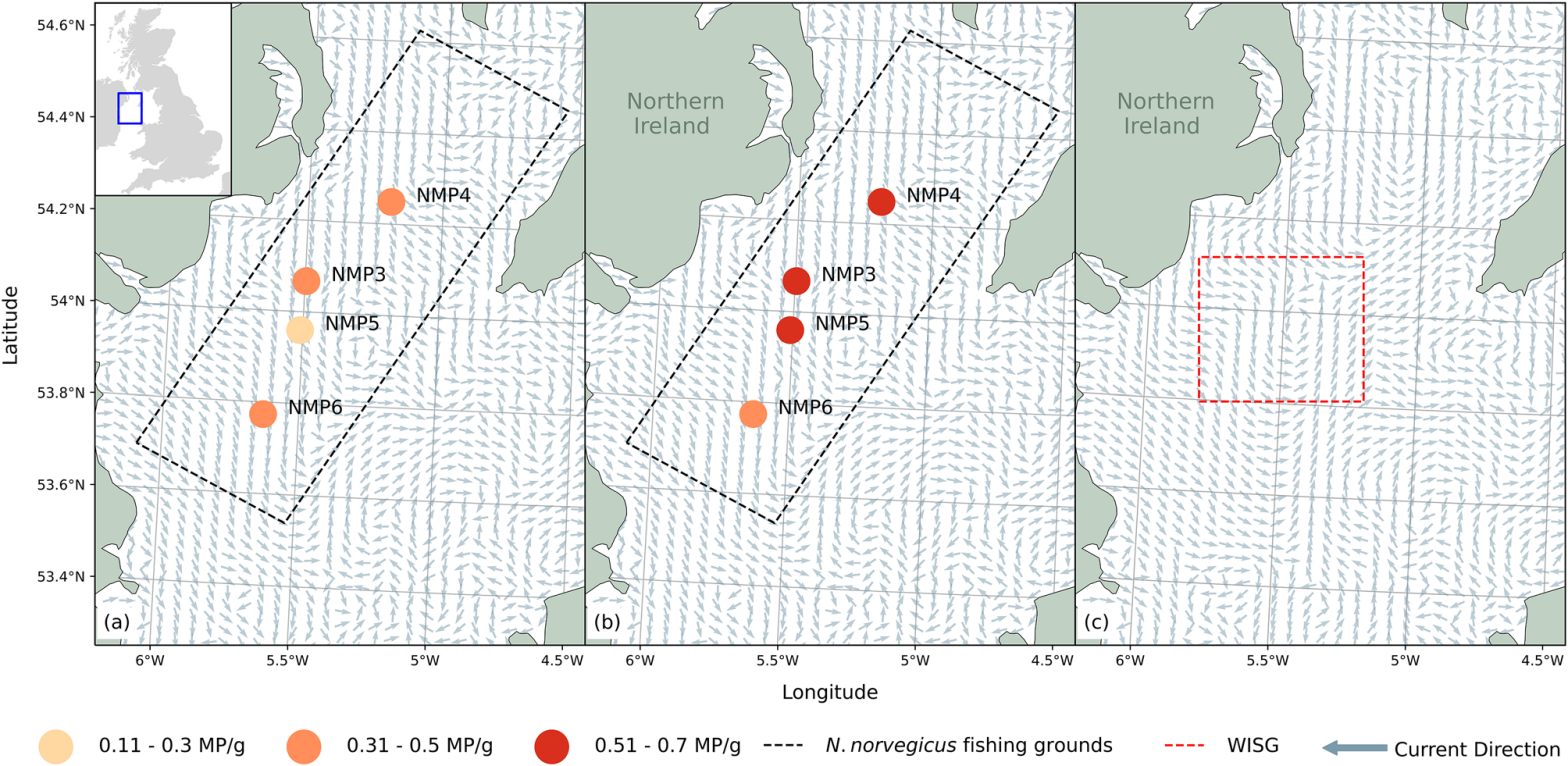
The location, site ID and mean microplastics per gram (MP/g) found within the four sediment sampling sites from the Western Irish Sea fishing grounds in (**a**) January 2016 and (**b**) January 2019. Monthly modelled residual currents for January 2020 (**a**,**b**) and July 2020 (**c**) demonstrating the development of the WISG in early summer. Modelled current data from E.U. Copernicus Marine Service Information^25^.

Previous studies of the Western Irish Sea have highlighted the presence of a cyclonic gyre referred to as the Western Irish Sea Gyre (WISG; Fig. 1c)^19^. Formation of the WISG is triggered by a strong thermocline trapping a cool dome of water where horizontal density gradients on each side drive cyclonic flow^20^. Gyres are known to accumulate organic and non-organic matter, and the physical conditions of the WISG promotes retention of *N. norvegicus*, and prevents any southward advective losses of larvae in early spring^21^. However, microplastics are known to accumulate within oceanic gyres e.g. most famously in the North Pacific Gyre^22^. Whether microplastic accumulates and settles within the WISG is currently unknown, however, due to the significance of the gyre in larvae retention and fecundity it is important to understand the dynamics of microplastic pollution in the region to help mitigate risks to regional species.

Here, we identified the abundance of microplastic pollution in the Western Irish Sea fishing grounds through density separation and digestion of 24 benthic sediment samples from four key sites in both 2016 and 2019. We hypothesised that microplastic abundance would increase across sample years, and also that microplastic pollution would accumulate in higher quantities closer to the Irish Sea gyre. Through µFTIR identification, we aimed to determine the abundance and type of microplastic pollution in the Western Irish Sea and highlight the potential implications of microplastic accumulation for the economically important *N. norvegicus* fishery.

## Methods

### Sediment sampling

Sediment grabs were conducted as part of a monitoring program for contaminants at four sites within the Western Irish Sea in January 2016 and January 2019 (*n* = 8 total). All samples were collected during research cruises on the RV *Corystes* at a range of depths from 68 to 167 m during January of each year (Fig. 1a,b; see Supplementary Table 1 for site coordinates and depths). Once collected, the sediment samples were stored in clean glass jars. The sampling sites were all located in an area known as the Irish Sea mud basin^23^ and had similar sediment characteristics which comprised of mostly < 63 µm silt and clay (~ 90%)^24^.

### Sample preparation & digestion

All eight sediment samples were labelled and subsequently dried on metal trays in an incubator at 40 °C. Once dried, the samples were gently ground with a pestle and mortar and sieved through a 2 mm sieve. The dried sediment was then divided into three representative subsamples using a Micro Riffler. Three subsamples from each site (total *n* = 24) were then added to 250 ml glass beakers and digested in 50 ml of pre-filtered 30% hydrogen peroxide (H_2_O_2_) at room temperature for 24 h to remove organic matter. After the digestate was then heated on a hotplate at ~ 50 °C for 30 min. The digestion process was then repeated using pre-filtered 2 M hydrochloric acid (HCL) to remove calcium carbonate from the sediment^26^.

### Density separation & filtration

The digested samples were twice washed with 45 mL filtered deionised water in a centrifuge at 3000 rpm for 3 min. Following this, 30 ml of a high density solution of Sodium Polytungstate (SPT; 1.6 s.g)^27^ was added and subsequently centrifuged at 3000 rpm for 20 min. Finally, the supernatant was decanted and subsequently vacuum filtered using a three piece Hartley pattern filter funnel and 25 mm VWR glass microfiber filter paper. Each filter paper was then dried at 40 °C and the remaining SPT solution was removed with a pipette and filtered for recycling.

### Visual and polymer identification

All filtrates were analysed under light microscopy initially using an Olympus SX16 stereoscope. Microplastic particles were categorised as either fibres, fragments, films, or spheres. Once identified visually, the microplastics were measured and photographed using a digital microscope (VHX-2000, Keyence, Osaka, Japan) before being transferred to aluminium oxide membrane filters (Whatman Anodisc filter; pore size 0.2 μm; diameter 47 mm) for subsequent spectroscopical analysis. For polymer verification, a subsample of the identified particles, identified by eye as representing the range of MP found in the samples, were analysed manually using a Fourier-transform infrared microscope (µFTIR, Hyperion 2000, Bruker, Ettlingen, Germany)^26^. To ensure that a representative subsample of microplastics was identified for the µFTIR analysis, at least one particle per potential microplastic type and sample filter was chosen. In the cases where microplastic spheres were identified, their small size made it impossible for us to transfer them onto aluminium oxide membrane filters manually and obtain a µFTIR spectrum from them. Although we are confident they are of plastic origin due to their shape and artificial colour, we did not include them in this study. This was also the case for some small fragments. In addition, we considered multiple similar microplastic fragments in the same sample as one larger particle, as we could not rule out fragmentation of microplastics when grinding our samples (see “Sample preparation & digestion”). As a result, we identified a subsample of 35% of the microplastics extracted via µFTIR.

### Contamination protocol

During the sample collection, all sediment samples were collected and stored in clean glass jars. Aluminium foil was used to cover samples at all times during the analysis. In the case that plastic items were used, they were prewashed twice using filtered deionised water. All work benches and laboratory equipment were washed using deionised water and inspected visually for airborne contamination before and between each stage of the analysis. In addition, to further reduce potential contamination, windows were permanently sealed and only a limited number of researchers were permitted in the laboratory. Further to this, 100% cotton laboratory coats and nitrile gloves were worn at all times. Additionally, natural fibre clothing was worn under laboratory coats throughout the analysis. Alongside the sediment samples, procedural blanks (i.e. purified and pre-sieved sand of equal weight to the subsamples) were used to quantify any contamination throughout the subsampling, digestion, and filtration stages. The procedural blanks were digested, separated, filtered, and inspected in the same way as the environmental samples. Atmospheric blanks were left open during each stage of the analysis to quantify airborne contamination in the laboratory. Both procedural and laboratory blanks were quantified for microplastic pollution and accounted for during the analysis^28^.

### Statistical analysis

A generalized linear model (GLM) assuming quasi-Poisson error distribution (owing to residual overdispersion) was used to determine differences among the microplastic counts across the four study sites, and between sampling years. All statistical analyses were carried out using the software program R v3.4.4^29^.

## Results

**Figure 2:**
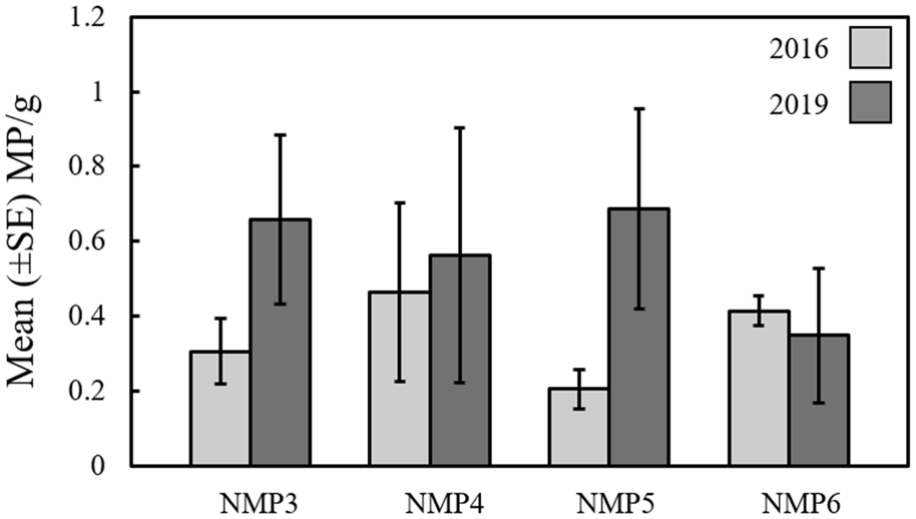
The mean (± SE) number of microplastics per gram of sediment at each site (NMP3–6) during research cruises in 2016 and 2019 in the Western Irish Sea.

A total of 77 individual microplastics were extracted from the Irish Sea sediment grabs in 2016 (30; 39%) and 2019 (47; 61%). The identified microplastics were categorised into two groups; fragments (71%; 55/77) and fibres (29%; 22/77). The mean (± SE) length of fragments and fibres were 154 ± 29 µm and 970 ± 202 µm, respectively. No significant difference was found in the total number of microplastics per gram (MP/g) identified between sampling years (F = 2.59, df = 19, *P* = 0.12), or among individual sites (F = 0.17, df = 20, *P* = 0.90; Fig. 2) and there was no significant “year x site” interaction effect (F = 0.88, df = 16, *P* = 0.47; Fig. 2).

Using µFTIR, a representative subsample of 27 candidate microplastic particles (35%) was used to visually identify the remaining particles. All particles counted as MP had a distinctive appearance, such as fibres of equal thickness and artificial colours^30^. Through µFTIR classification, all candidate microplastics were identified as man-made. Seven different polymers were identified; Acrylic/Alkyd (3), Epoxy resin (1), Polyacrylonitrile (5), Polyester (12), Polyethylene (1), Polypropylene (4), and Polystyrene (1). A small number of natural particles were also identified via µFTIR (8) (See Supplementary figure for µFTIR spectra). We considered some MPs as varnish particles because they consisted of characteristic polymer types such as acrylic/alkyd and epoxy resin (and polyester) and were chip like or resembled varnish which was previously found in other studies^31^.

The procedural blanks contained a mean (± SE) of 0.03 ± 0.03 and 0 of suspected synthetic particles per gram of sediment in 2016 and 2019, respectively. One polyester fibre was identified in the blanks using µFTIR, however, it was visibly different from other polyester fibres identified in the sediment samples and therefore no adjustments to the results were made. An additional 19 natural particles (16 fibres & 3 fragments) were also visually identified. From the confirmed polymer types, we inferred that potential varnish particles made up 13% (10/77) of the total microplastics.

## Discussion

Here, we show that microplastic abundances in the Irish Sea fishing grounds did not differ across sampling years, despite the increase in global plastic production between 2016 and 2019^1^. However, our study reports benthic microplastic sediment loads that range from 0–1.17 MP/g (Sup Table 1). Although there was a relatively low sample size, 83% (22/24) of subsamples contained microplastic pollution, highlighting the extent of microplastic contamination within this commercially important fishing area. The direct microplastic counts found in our study are comparable with microplastic abundances found in other systems such as the Mediterranean Sea (0.18–0.64 MP/g)^32^, the North Sea (0.002–1.18 MP/g)^33^, and the Atlantic Ocean (0.10 MP/g)^34^, although the extraction methodologies differed among studies.

We also found that microplastic accumulation was not influenced by the presence of the WISG in this study as the abundance of microplastics at site were not statistically different. This may be related to low sampling resolution as only four sites were tested across two individual sampling years. Additionally, the WISG develops during late spring and is strongest during the Summer months^21^. This study’s samples were collected in January which may explain why there was no clear pattern of higher microplastic accumulation towards the centre of the WISG.

It is also possible that the high level of variance found among the sites was the reason we did not find a pattern of microplastic abundance. All sampling sites from the *N. norvegicus* fishing grounds were located within the Irish Sea mud basin area, and therefore were very similar in terms of sediment characteristics. Previous studies have shown how the accumulation of microplastics can vary in accordance to sediment types^26,35^.

The identified microplastics within the samples are suspected to have come from a range of sources. Exact sources are impossible to detect but the acrylic/alkyd, epoxy resin and polyester microplastic fragments could have been derived from weathering ship paint^26,36^. We are certain that these paint fragments did not derive from our research ship as its external coating was black and white in colour as opposed to the blue and red fragments we found in the study. In contrast, polyester and polyacrylonitrile fibres most likely stem from textiles^37^ while polyethylene is a typical material for plastic shopping bags^38^ and general packaging^1^. Similarly, polypropylene^39^ and polystyrene^40^ are used for single use products such as food packaging. Hence, the microplastics in our samples are presumably derived primarily from land (clothing, food packaging, and cosmetics)^41^ and some from ocean (ship paint) based sources.

Microplastic particles are known to act as vectors for the transport and transference of Endocrine Disrupting Compounds (EDCs)^42^ in marine systems including persistent organic pollutants (POPs)^43^. Particularly, bisphenol A (BPA), a compound that strengthens some of the plastic polymers found in our samples, such as epoxy resin and polyester, is associated with a range of adverse effects on human health such as cancers, infant developmental issues, and female reproductive issues^44^. These pollutants can also be passed through marine food-webs and bioaccumulate in top predators^45^. There is also a growing concern that EDCs can be transferred to humans through the consumption of seafood^42^, which is alarming when considering how commercially important the Irish Sea *N. norvegicus* industry is. Other types of EDCs such as plasticisers and antioxidants can leach from the polymer into the surrounding water^42^ highlighting that marine species could be exposed to these pollutants without ingesting microplastic particles.

It is likely that *N. norvegicus* passively ingest microplastics with sediment as they are feeding as it is common for non-food items such as sediment to be found in their stomachs^46^. It has also been shown that *N. norvegicus* actively move sediment around their mouthparts which may be a mechanism for ingesting smaller attached organisms^47^. It is therefore likely that microplastic uptake from the sediment surrounding their burrows is a common ingestion pathway^9^. However, when fed microplastic fibres under laboratory conditions at representative environmental levels^9^, *N. norvegicus* individuals were shown to lose ~ 0.02% of their body mass each day throughout an 8 month feeding experiment^9^. This equates to a ~ 4.8% reduction in body mass across the duration of the study in total. When considering the levels of microplastic pollution identified within this study, it is possible that microplastics will be ingested by *N. norvegicus*^18^, and could lead to a negative effect on their body mass^9^ and therefore the sustainability of the fishery in future. Although purely speculative, if one was to consider a ~ 4.8% reduction in *N. norvegicus* body mass could lead to a ~ 4.8% reduction in fisheries landings, current levels of microplastic pollution in the Irish Sea may have cost the UK economy ~ £3 million in 2020 alone (based on UK *N. norvegicus* landings in 2020 valued at £63 million)^48^.

In conclusion, this study highlights the consistent presence of microplastics in a commercially important fishing area. However, as microplastic accumulation did not increase across sampling years, and was not influenced by the WISG in this instance, further research is needed to determine the factors that drive microplastic accumulation in the Irish Sea and other such commercially important fishing grounds which include the role of currents and sediment characteristics. As microplastic ingestion is commonly found in *N. norvegicus*^18^, and can result in a reduction of body mass^9^, we suggest that the particles found in this study could be ingested and may lead to adverse effects on physiology. This may lead to negative implications for the sustainability of the Western Irish Sea *N. norvegicus* fishery in future.

## Supplementary Information

Supplementary Information.
