# Author Correction: The accumulation of microplastic pollution in a commercially important fishing ground

**DOI:** 10.1038/s41598-022-09956-6

**Published:** 2022-04-06

**Authors:** Eoghan M. Cunningham, Sonja M. Ehlers, Konstadinos Kiriakoulakis, Pia Schuchert, Nia H. Jones, Louise Kregting, Lucy C. Woodall, Jaimie T. A. Dick

**Affiliations:** 1 Queen’s University Marine Laboratory, Queen’s University Belfast, 12‑13 The Strand, Portaferry, BT22 1PF Northern Ireland UK; 2 School of Biological and Environmental Sciences, Liverpool John Moores University, 3 Byrom St, Liverpool, L3 3AF UK; 3 Department of Zoology, University of Oxford, 11a Mansfeld Road, Oxford, OX1 3SZ UK; 4 Department of Animal Ecology, Federal Institute of Hydrology, Am Mainzer Tor 1, 56068 Koblenz, Germany; 5 Institute for Integrated Natural Sciences, University of Koblenz-Landau, 56070 Koblenz, Germany; 6 Agri-Food and Biosciences Institute, 18a Newforge Lane, Belfast, BT9 5PX Northern Ireland UK; 7 School of Ocean Sciences, Bangor University, Anglesey, LL59 5AB UK; 8 School of Natural and Built Environment, Queen’s University Belfast, Belfast, BT9 5BN UK; 9 Nekton Foundation, Oxford, OX5 1PF UK

Correction to: *Scientific Reports* 10.1038/s41598-022-08203-2, published online 10 March 2022

The original version of this Article contained an error in Fig. 1 where the values were incorrect. The original Fig. 1 and accompanying legend appear below.

**Figure 1:**
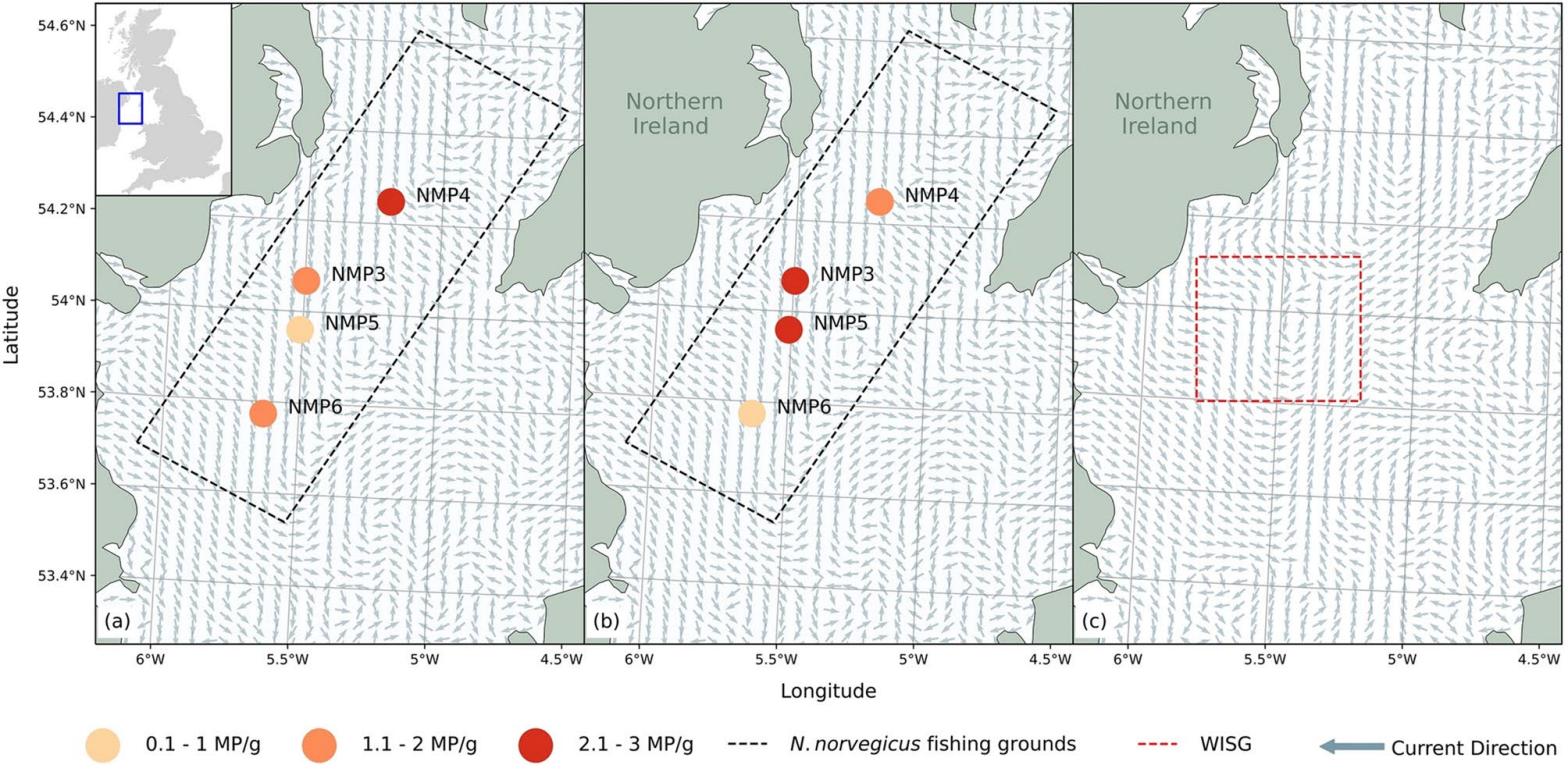
The location, site ID and mean microplastics per gram (MP/g) found within the four sediment sampling sites from the Western Irish Sea fishing grounds in (**a**) January 2016 and (**b**) January 2019. Monthly modelled residual currents for January 2020 (**a**,**b**) and July 2020 (**c**) demonstrating the development of the WISG in early summer. Modelled current data from E.U. Copernicus Marine Service Information^25^.

The original Article has been corrected.

