# Supplementary material for: The accumulation of microplastic pollution in a commercially important fishing ground
Source: Sci Rep. 2022 Mar 10;12:4217. doi: 10.1038/s41598-022-08203-2 (PMC8913702; doi:10.1038/s41598-022-08203-2)
Supplement: Supplementary file 1 — Supplementary Information. [file 41598_2022_8203_MOESM1_ESM.docx]

**The accumulation of microplastic pollution in a commercially important fishing ground**

Eoghan M. Cunningham^1,2,3^*†, Sonja M. Ehlers^4^†, Konstadinos Kiriakoulakis^2^, Pia Schuchert^5^, Nia H. Jones^6^, Louise Kregting^1,7^, Lucy C. Woodall^3,9^, Jaimie T. A. Dick^1^

**Supplementary Material**

**Supplementary Table S1:** The locations of the four sediment sampling sites from the Western Irish Sea fishing grounds. Data includes the Site ID, Latitude, Longitude, and Depth (m).

| **Site ID** | **Latitude** | **Longitude** | **Depth (m)** |
| --- | --- | --- | --- |
| NMP3 | 54.067267 | -5.496467 | 68 |
| NMP4 | 54.248617 | -5.194767 | 145 |
| NMP5 | 53.959833 | -5.510833 | 167 |
| NMP6 | 53.773617 | -5.631833 | 155 |


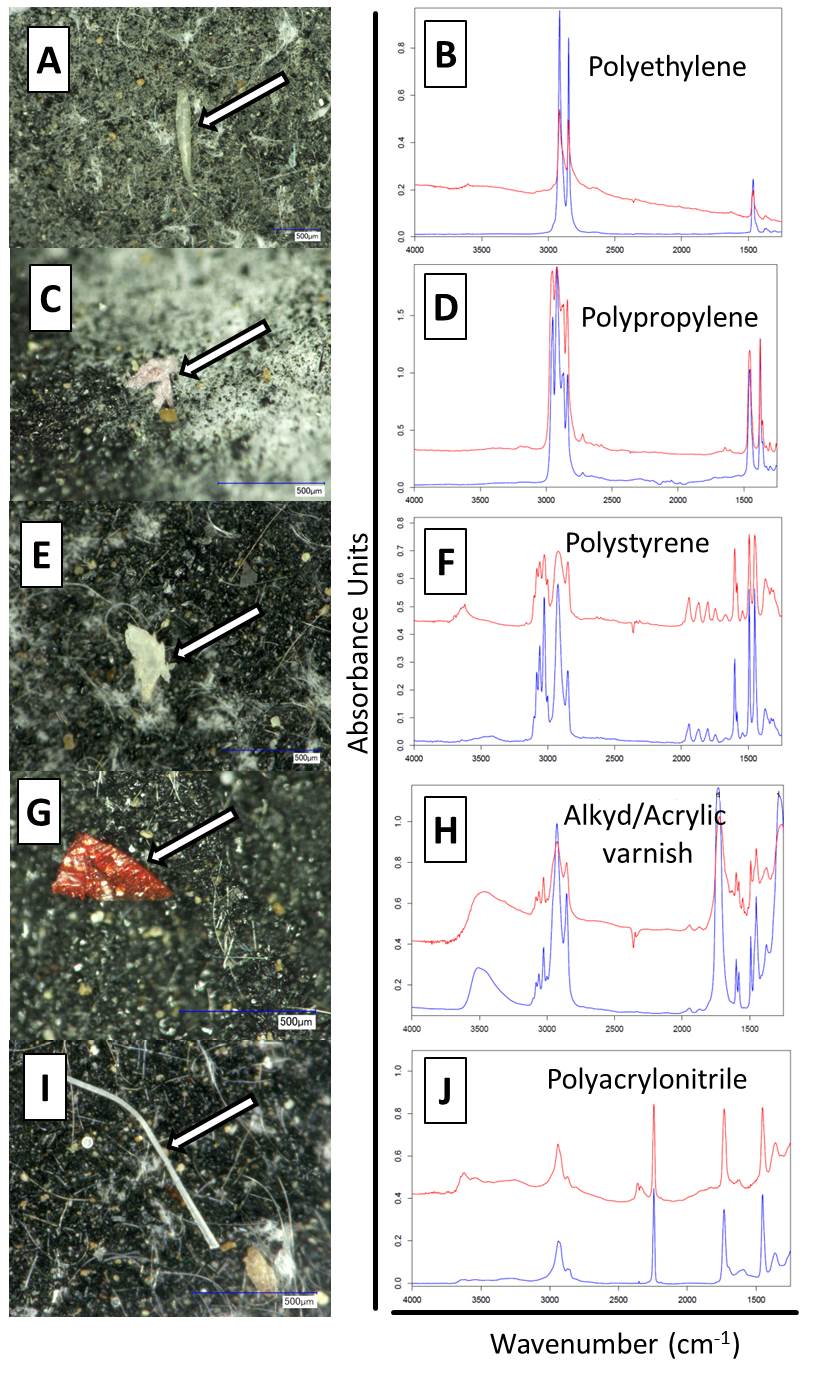


**Supplementary Figure S1:** Plastic particles (A, C, E, G, I) with their corresponding µFTIR spectra (B, D, F, H, J; red) and the reference library spectra (blue). H) The reference spectrum is styrenated alkyd. µFTIR measurements were conducted in transmission mode.
